# Hotspots and trends in liver kinase B1 research: A bibliometric analysis

**DOI:** 10.1371/journal.pone.0259240

**Published:** 2021-11-04

**Authors:** Yaowen Song, Fangkun Zhao, Wei Ma, Guang Li

**Affiliations:** 1 Department of Radiotherapy Oncology, The First Affiliated Hospital of China Medical University, Shenyan, China; 2 Department of Ophthalmology, The Fourth Affiliated Hospital of China Medical University, Shenyang, China; 3 Department of Breast Surgery, The First Affiliated Hospital of China Medical University, Shenyang, China; Northumbria University, UNITED KINGDOM

## Abstract

**Introduction:**

In the past 22 years, a large number of publications have reported that liver kinase B1 (LKB1) can regulate a variety of cellular processes and play an important role in many diseases. However, there is no systematic bibliometric analysis on the publications of LKB1 to reveal the research hotspots and future direction.

**Methods:**

Publications were retrieved from the Web of Science Core Collection (WoSCC), Scopus, and PubMed databases. CiteSpace and VOSviewer were used to analysis the top countries, institutions, authors, source journals, discipline categories, references, and keywords.

**Results:**

In the past 22 years, the number of LKB1 publications has increased gradually by year. The country, institution, author, journals that have published the most articles and cited the most frequently were the United States, Harvard University, Prof. Benoit Viollet, Journal of Biochemistry and Plos One. The focused research hotspot was the molecular functions of LKB1. The emerging hotspots and future trends are the clinical studies about *LKB1* and co-mutated genes as biomarkers in tumors, especially in lung adenocarcinoma.

**Conclusions:**

Our research could provide knowledge base, frontiers, emerging hotspots and future trends associated with LKB1 for researchers in this field, and contribute to finding potential cooperation possibilities.

## Introduction

LKB1 (liver kinase B1), also known as STK11 (serine/threonine kinase 11), is a protein kinase encoded by the *STK11* gene in humans. LKB1 is widely expressed in various tissues, and the expression level is highest in testis and fetal liver [1]. LKB1 is considered as a “master kinase” that regulates various cellular processes, including metabolism, differentiation, polarity, division, proliferation, migration, apoptosis and DNA damage response [2–6]. Given its wide range of biological functions, thousands of articles have been published reporting the regulatory mechanism of LKB1 in a variety of physiological and pathological processes, including malignancies, metabolic disease, cardiogenic diseases, skeletal muscle and development, and angiogenesis, etc. [2, 3, 7–12]. Recently, preclinical studies and clinical trials where *LKB1* mutation was among the primary and secondary inclusion criteria have been conducted successively [13–17]; however, screening for *LKB1* mutation has not been routinely applied in the clinic, as controversies and unexplored aspects of LKB1 activity remain. Therefore, investigations of LKB1 have important medical implications that require in-depth analysis and summary.

Bibliometric analysis is an effective mathematical and statistical method in summarizing hotspots and emerging trends in specific scientific fields, through quantitative analysis of related scientific literature. Mapping knowledge domains of bibliometric are metrological methods applied to determine the structures, rules, distributions, characteristics and research frontiers of a scientific discipline in a visual way, using statistics, graph theory and computer technology. VOSviewer and CiteSpace are effective visualization software tools which apply the mapping knowledge domains [18–20]. Both software had been recognized by scientists. The statistics analyzed by the two software were used by scientists to publish a large number of articles in fields such as medicine, molecular biology, agriculture, and environmental science, etc. [21–27]. These articles provided scholars with a wealth information of core research power, hotspots, and global trends in their respective fields.

Currently, a bibliometric analysis on LKB1 research has not been published. Based on the advantages of bibliometric analysis software VOSviewer and CiteSpace, our research makes a bibliometric analysis of LKB1 related publications, and reveals the core research power, hotspots evolution and future trends of LKB1 research.

## Materials and methods

### Search strategy

The publications used for bibliometric analysis were downloaded in June 1, 2021, from the multidisciplinary citation databases Web of Science Core Collection (WoSCC) and Scopus, as well as the life sciences and biomedical disciplines database PubMed. The search criteria were as following: search topics, “STK11” or “serine-threonine kinase 11” or “LKB1” or “liver kinase B1”; document type, “article”; year range, “2000 to 2021”; and no limit on language was set. The search strategies of the three databases were listed in S1 File.

### Data preprocessing

All the publications downloaded from WoSCC, Scopus and PubMed were imported into Endnote X9 for deduplicating. Due to different versions of spelling in the title or author, we further carried out manual deduplicating. After deduplicating, two researchers conducted a screening to exclude the publications that did not met the research topic and search strategy, as well as those that had not been reviewed by peers or had been withdrawn, so as to improve the quality of the included publications.

The multi-databases combined bibliometric analysis could be performed only when the formats of data downloaded from deferent databases were unified. We used the format conversion function of Citespace5.7.R5W to convert the format of the data downloaded from Scopus and PubMed into WOS format, as same as the data downloaded from WoSCC. Then, two researchers revised the data with WOS format through adding the missing value and modifying the error codes (S1 Dataset).

### Data analysis

VOSviewer1.6.16, which was developed by Van Eck and Waltman of Leiden University, has the advantages of conducting accurate statistical analysis, clustering large-scale data, generating density visualization, and locating scientific research hotspots [18]. In this study, we selected citation, co-citation, co-authorship and co-occurrence analysis to report and classify the top countries, institutions, authors, source journals, cited references, keywords of the retrieved publications. Furthermore, we chose network views to map the core research power and their collaborative relationships, as well as the evolution of hotspots. The parameters of VOSviewer were setting as following: the minimum number of publications per author, country, organization and source journal was 5; the minimum co-citation frequency of each cited reference was 20; the minimum co-occurrence frequency of each keyword was 5; the minimum size of each cluster was 1; the random starts parameter was 10; the number of iterations were 10; and the random seed parameter was 0. In the network view map, bigger node size represents greater number of publications; shorter lines represent closer collaborations; and the nodes with the same color represent a cluster that have similar research theme. The detailed explanations are provided in the manual of VOSviewer on line (https://www.vosviewer.com/documentation/Manual_VOSviewer_1.6.16.pdf).

CiteSpace5.7.R5W, which was developed by Chaomei Chen of Drexel University, is another commonly used bibliometrics software [20]. We used its data processing utilities and category analysis function to generate the annual number of publications and top discipline categories, respectively. Then, GraphPad Prism 9 was used to present the publication trends and top 10 discipline categories in line chart and bar chart, respectively. CiteSpace has a special analysis method, namely citation-burst-time analysis, which is used to identify the time point when a certain research direction becomes a hotspot [19, 28]. We analyzed the keywords using the citation burst history, which can quantify burst strength and locate burst time, so as to estimate developing trends quantitatively. The list of strongest citation burst keywords was mapped by minimum tree generation algorithm. The parameters of citation burst analysis were setting as following: the configure detection model is f(x) = αe^-αχ^, α_1_/α_0_ = 2.0, α_i_/α_i-1_ = 2.0; the number of states is 2, γ [0,1]; and minimum duration is 2. In the time line map, the red segment on the green timeline represents the begin and end years between which the keyword had been burst cited, and the “strength” represents the strength with which the keyword had been burst cited. The manual of CiteSpace is available on website (http://cluster.ischool.drexel.edu/∼cchen/citespace/CiteSpaceManual.pdf).

## Results

### Description and trends of publications

**Fig 1:**
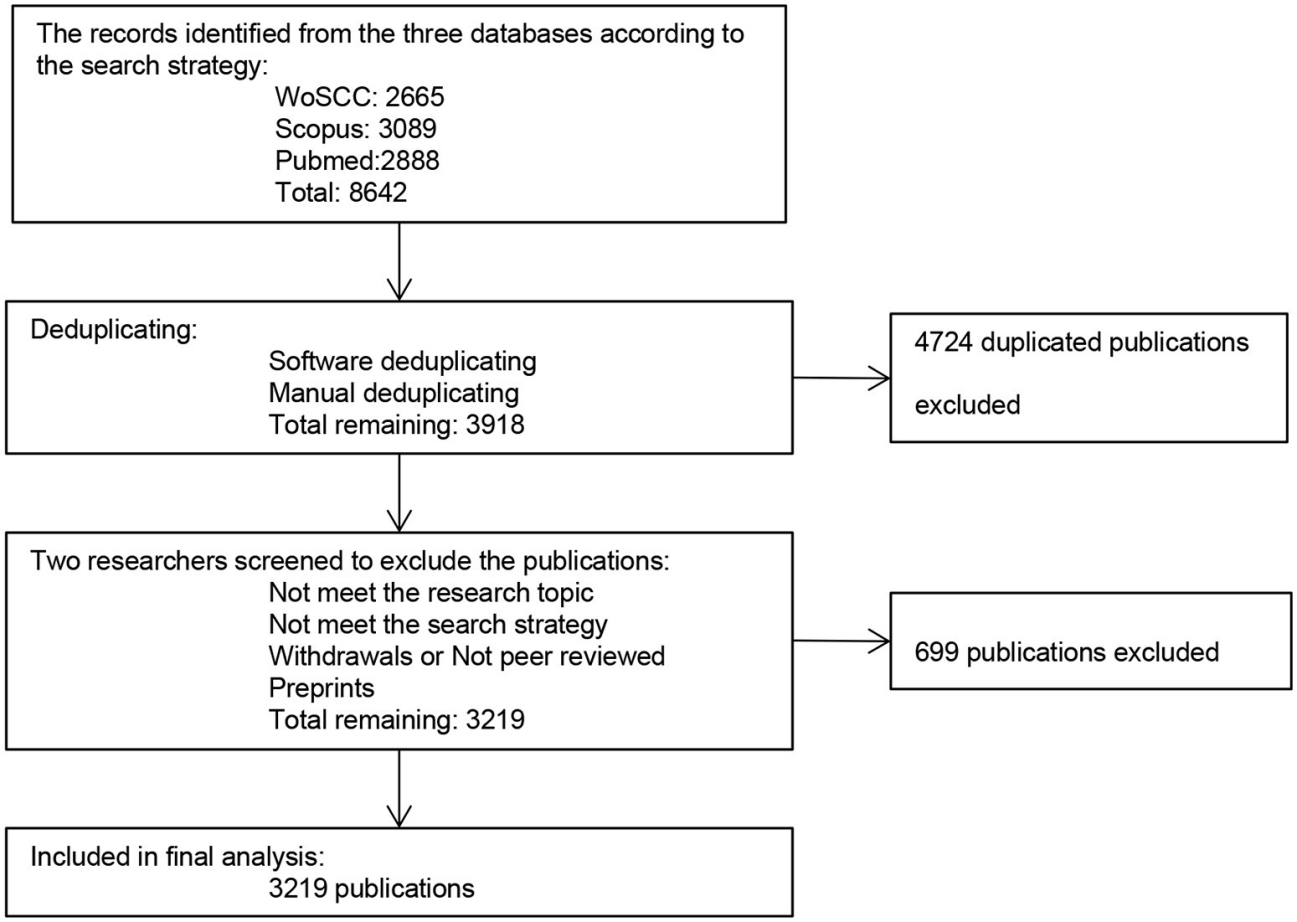
Flow chart of LKB1 researches inclusion.

**Fig 2:**
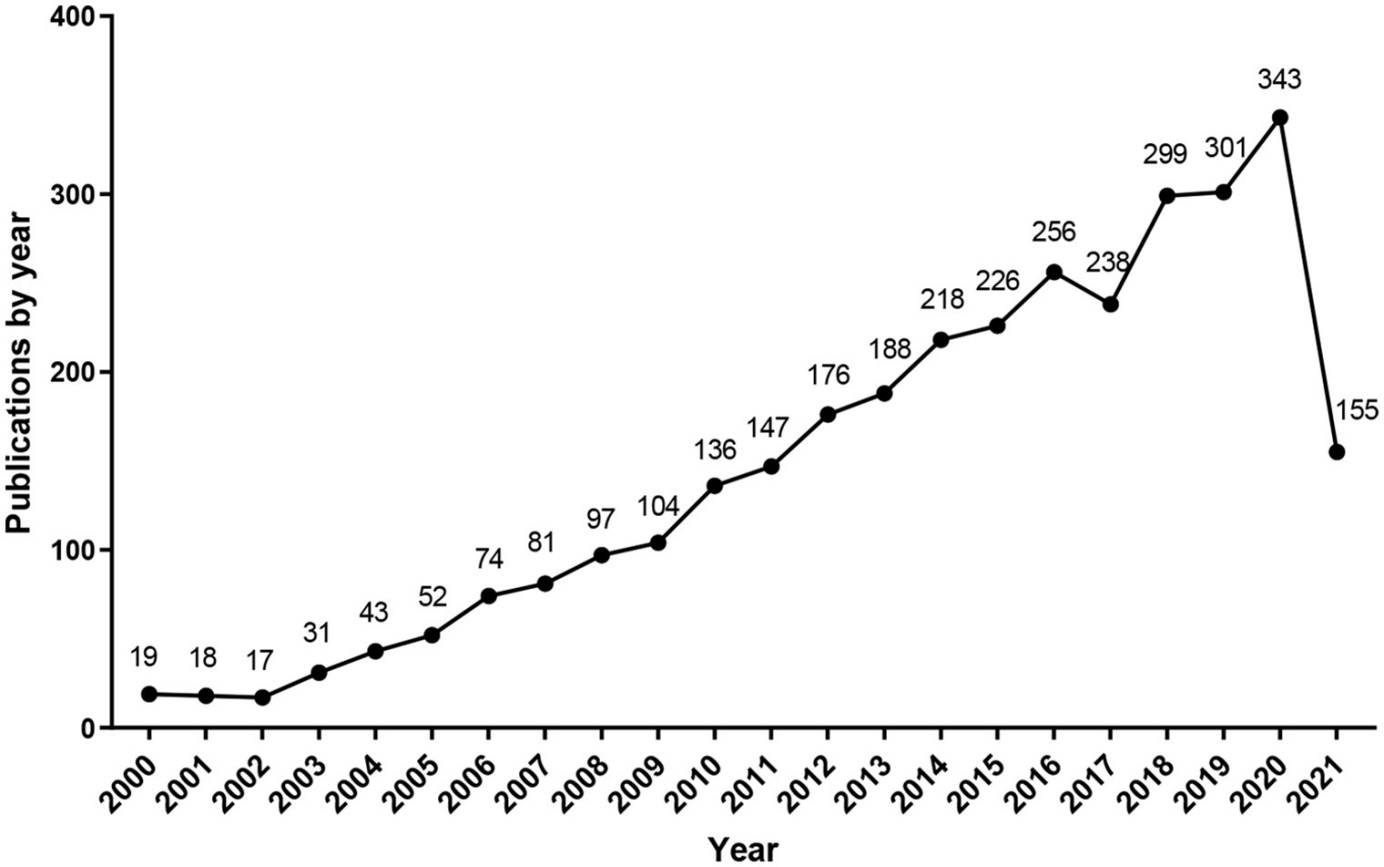
Distribution of publications by years.

A total of 8,642 publications were extracted, WoSCC (2,665 publications), Scopus (3,089 publications), PubMed (2,888 publications). After data preprocessing, 3,219 publications were retrieved for final analysis (Fig 1). To visualize the growth trend of LKB1 research, we generated a line chart according to the annual number of publications. As shown in Fig 2, the number of publications increased gradually, with a peak in 2020. From 2016 to 2021, the number of publications accounted for 49.46% of the total.

### Discipline categories

**Fig 3:**
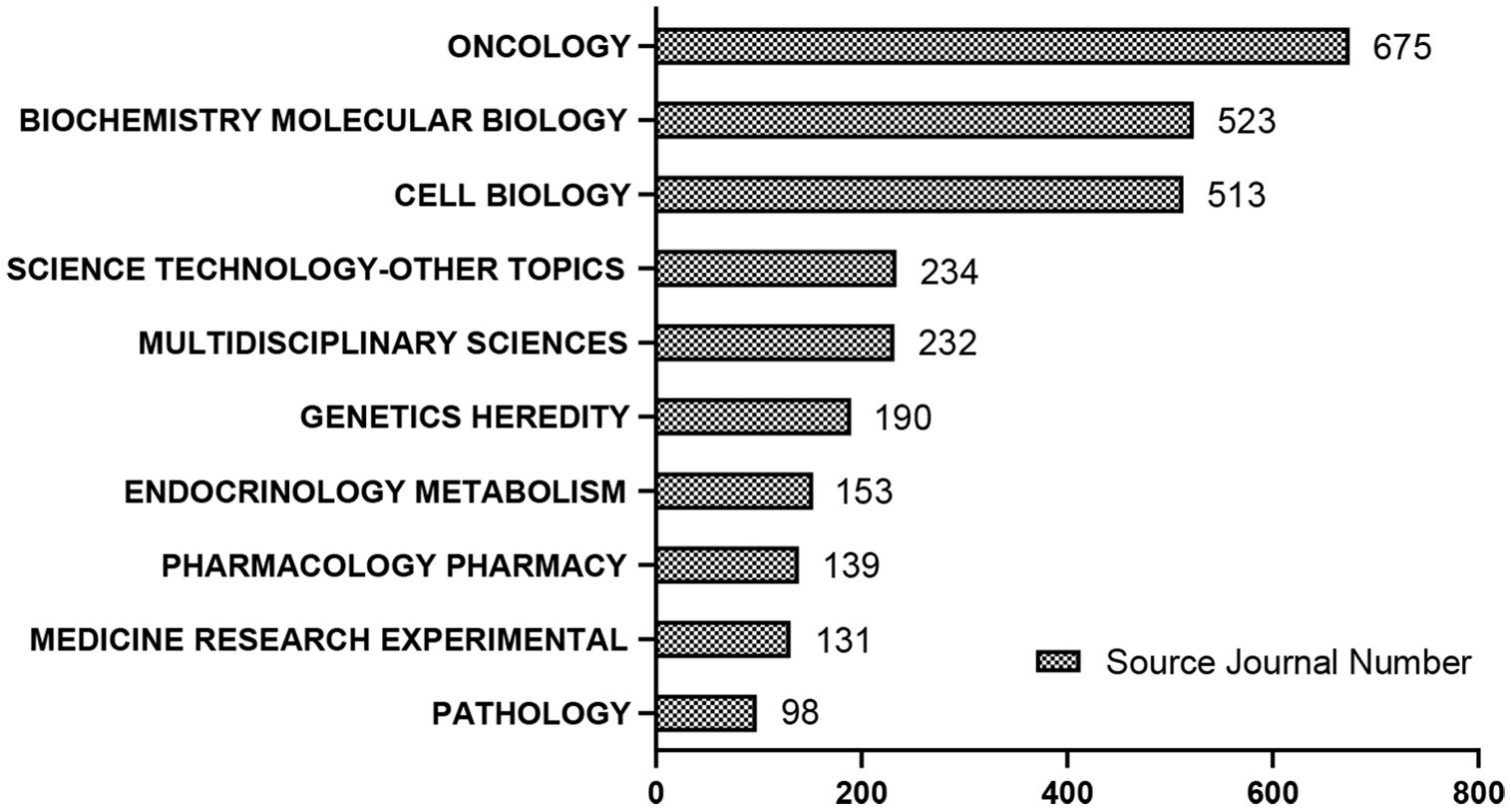
Top 10 discipline categories in LKB1 research.

Based on the scientific attributes of the retrieved data, the discipline categories of LKB1 research mainly focused on oncology (675 publications), biochemical molecular biology (523 publications), and cell biology (513 publications) (Fig 3).

### Country and institution analysis

According to the data of countries citation analysis by VOSviewer, the United States published the largest number of publications on LKB1 (1,018 publications, 31.62%), followed by China (714 publications, 22.18%) and South Korea (248 publications, 7.70%). The country with the highest number of citations was the United States with 77,696 (Table 1).

**Table 1:** Top 10 productive countries in LKB1 research, 2000 to 2021.

Rank	Country	Count	Percentage (%)	Citation	Average year of publication
1	United States	1018	31.62	77696	2014.70
2	China	714	22.18	16556	2017.02
3	South Korea	248	7.70	9824	2014.84
4	Japan	226	7.02	7550	2013.32
5	France	186	5.78	12338	2014.04
6	Canada	153	4.75	9766	2013.78
7	England	145	4.51	12050	2011.58
8	Germany	136	4.22	7215	2013.68
9	Italy	132	4.10	5203	2015.24
10	Spain	113	3.52	4932	2013.11

**Fig 4:**
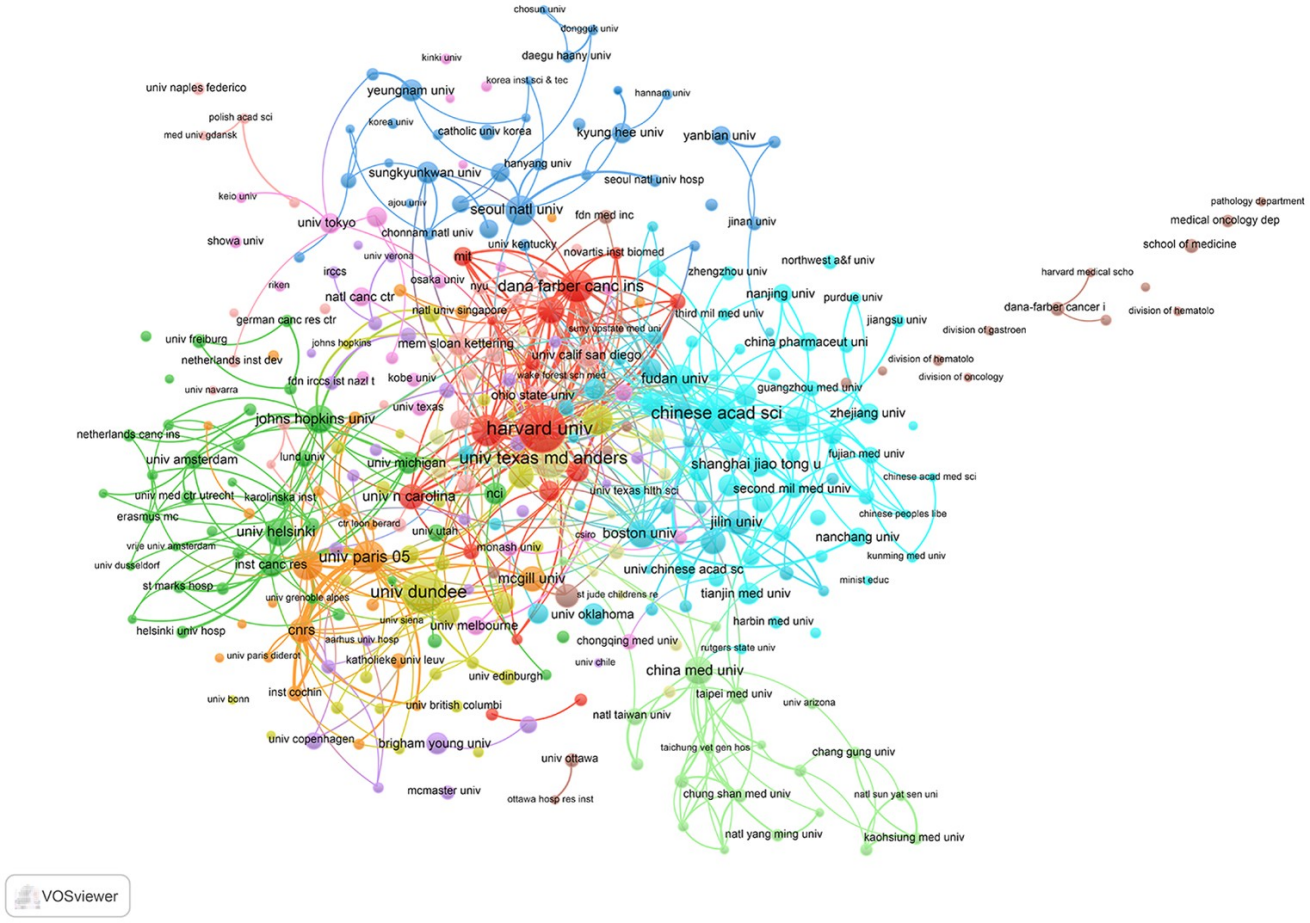
Collaboration network of main institutions.

The institution that published the largest number of publications was Harvard University (101 publications, 3.14%), followed by the University of Dundee (81 publications, 2.52%), and Chinese Academy of Sciences (71 publications, 2.21%), meanwhile, Harvard University also had the highest number of citations (1,5096 citations) (Table 2). Half of the most active institutions (top 10) were in the United States. A network view map was generated through co-authorship analysis, which enables us to visualize the collaborative network relationship between relevant research institutions (Fig 4). As shown in Fig 4, there was a complex collaborative relationship among the major institutions.

**Table 2:** Top 10 productive institutions in LKB1 research, 2000 to 2021.

Rank	Institution	Country	Count	Percentage (%)	Citation	Average year of publication
1	Harvard University	United States	101	3.14	15096	2011.05
2	University of Dundee	England	81	2.52	12828	2009.57
3	The University of Texas MD Anderson Cancer Center	United States	71	2.21	4590	2015.49
4	Chinese Academy of Sciences	China	71	2.21	3454	2014.79
5	University Paris	France	54	1.68	4677	2013.56
6	Dana-Farber Cancer Institute	United States	51	1.58	8719	2013.57
7	Massachusetts General Hospital	United States	45	1.40	7008	2014.54
8	Inserm	France	43	1.34	4028	2011.98
9	Emory University	United States	43	1.34	1690	2013.93
10	Fudan University	China	41	1.27	2024	2014.34

### Top co-authorship authors analysis

**Fig 5:**
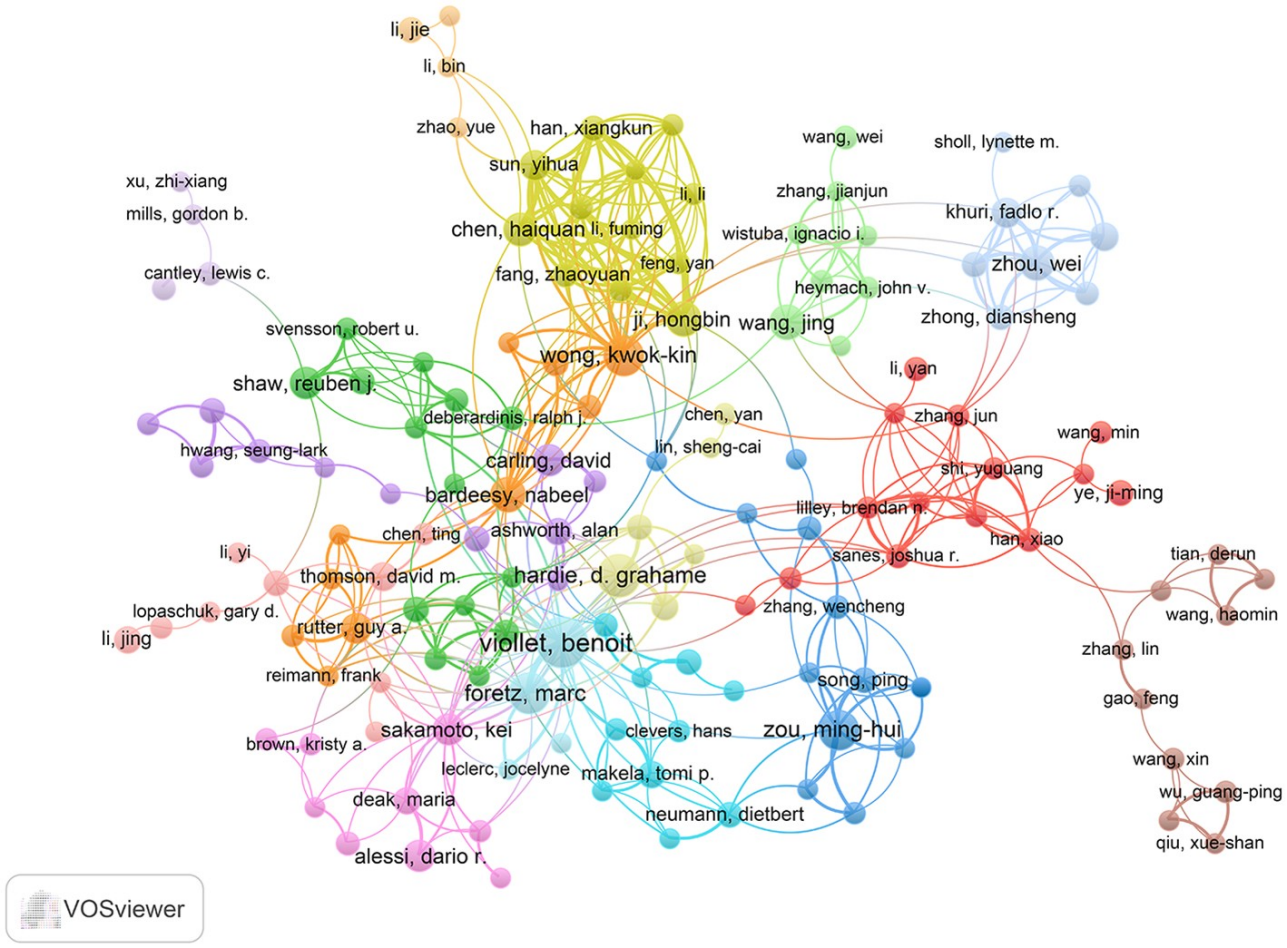
Co-authorship network of authors producing LKB1 research.

By using VOSviewer, the results of co-author analysis showed that a total of 20,649 authors participated in the publication of 3,219 LKB1 papers. The top 10 productive authors are listed in Table 3, Benoit Viollet (37 publications) ranked first, followed by D Grahame Hardie (25 publications) and Marc Foretz (23 publications). Moreover, the most cited authors were Benoit Viollet (4,081 citations), D Grahame Hardie (2,661 citations), and Kei Sakamoto (2,279 citations). The partnerships among the active authors were displayed by a network view map (Fig 5). The most active authors (top 10) had pronounced partnerships, for example, the links between Benoit Viollet, D Grahame Hardie, Marc Foretz, and Kei Sakamoto; between D Grahame Hardie, Kei Sakamoto, and Dario R Alessi; and between Kwok Kin Wong, Nabeel Bardeesy, and Hongbin Ji, etc. There were also several relatively independent research teams, such as Minghui Zou’s team, Jing Wang’s team, and Wei Zhou’s team, etc.

**Table 3:** Top 10 authors in LKB1 research, 2000 to 2021.

Rank	Authors with the highest publication outputs			Authors with the highest citations		
	Author	Country	Count	Author	Country	Citation
1	Benoit Viollet	France	37	Benoit Viollet	France	4081
2	D Grahame Hardie	England	25	D Grahame Hardie	England	2661
3	Marc Foretz	France	23	Kei Sakamoto	Switzerland	2279
4	Kwok Kin Wong	United States	21	Marc Foretz	France	1872
5	Minghui Zou	United States	21	David Carling	England	1859
6	Nabeel Bardeesy	United States	16	Dario R Alessi	England	1682
7	Hongbin Ji	China	16	Simon A Hawley	England	1476
8	Jing Wang	United States	16	Nabeel Bardeesy	United States	1456
9	Wei Zhou	United States	16	Kwok Kin Wong	United States	1391
10	Haiquan Chen	China	15	Russell G Jones	Canada	1231

### Citation analysis of source journals

**Fig 6:**
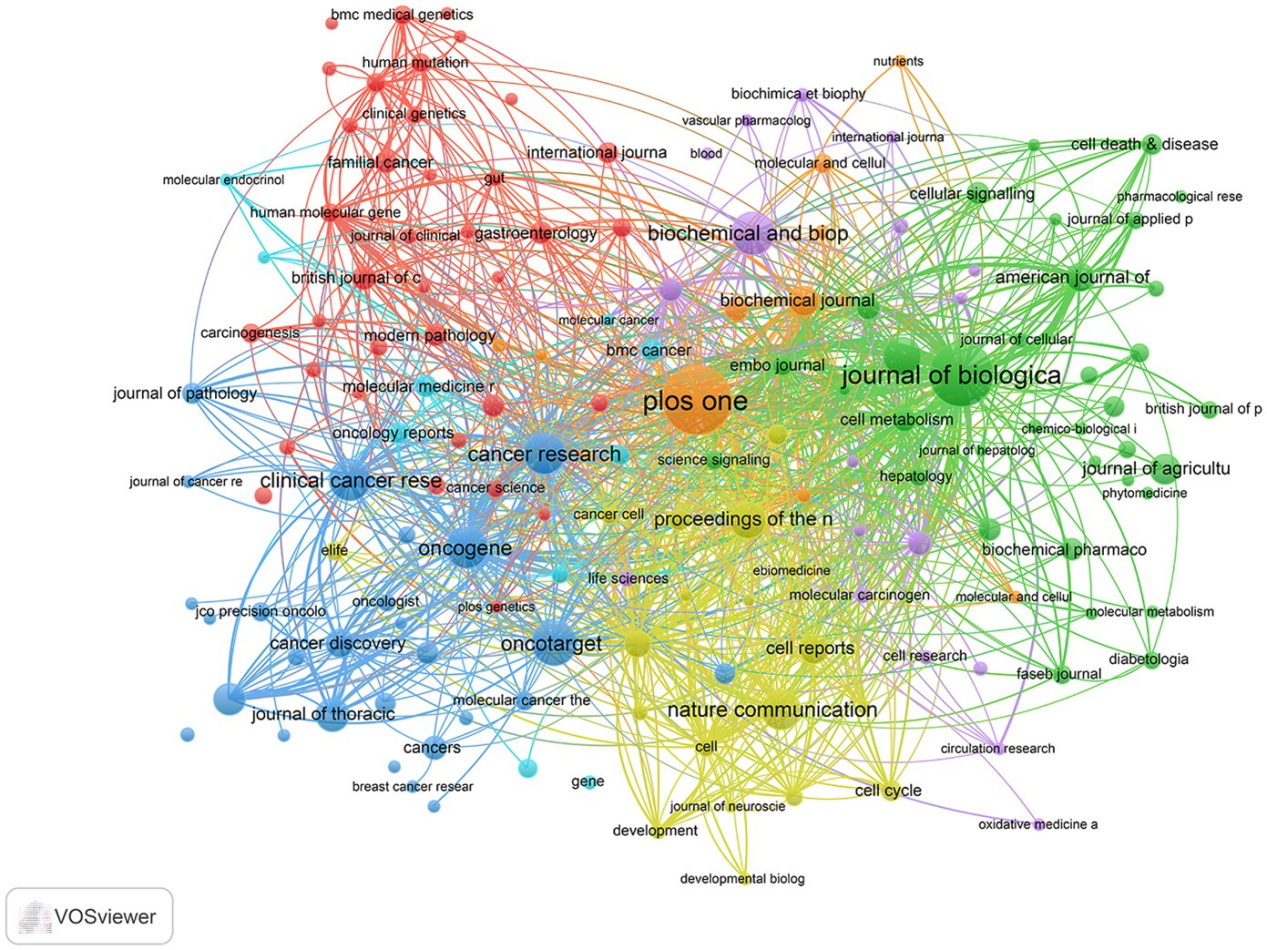
Network view of journals involved in LKB1 research.

There were 911 journals that contributed to the LKB1 related publications. The top 10 publication and citation journals are listed in Table 4, more than half of them belong to the United States. The most prolific journals were Plos One (108 publications) and Journal of Biological Chemistry (96 publications). Meanwhile, Journal of Biological Chemistry was the journals with the most cited number (8,970 citations), and Plos One ranked top 4 (4,951 citations). A network visualization was used to show leading journals and their clusters in different subject areas (Fig 6).

**Table 4:** Top 10 source journals for LKB1 articles, 2000 to 2021.

Rank	Journals with the highest publication outputs				Journals with the highest citations			
	Journal Title	Country	Count	Impact Factor (2020)	Journal Title	Country	Citation	Impact Factor (2020)
1	Plos One	United States	108	3.24	Journal of Biological Chemistry	United States	8970	5.157
2	Journal of Biological Chemistry	United States	96	5.157	Nature	England	6282	49.962
3	Oncotarget	United States	51	-	Proceedings of the National Academy of Sciences of the United States of America	United States	6005	11.205
4	Biochemical and Biophysical Research Communications	United States	48	3.575	Plos One	United States	4951	3.24
5	Oncogene	England	46	9.867	Cancer Research	United States	4351	12.701
6	Clinical Cancer Research	United States	45	12.531	Cell Metabolism	United States	4218	27.287
7	Cancer Research	United States	44	12.701	Science	United States	3825	47.728
8	Nature Communications	England	43	14.919	Cell	United States	3295	41.582
9	Scientific Reports	England	39	4.379	Embo Journal	United States	3030	11.598
10	Proceedings of the National Academy of Sciences of the United States of America	United States	36	11.205	Biochemical Journal	England	2918	3.857

### Co-cited reference analysis

**Fig 7:**
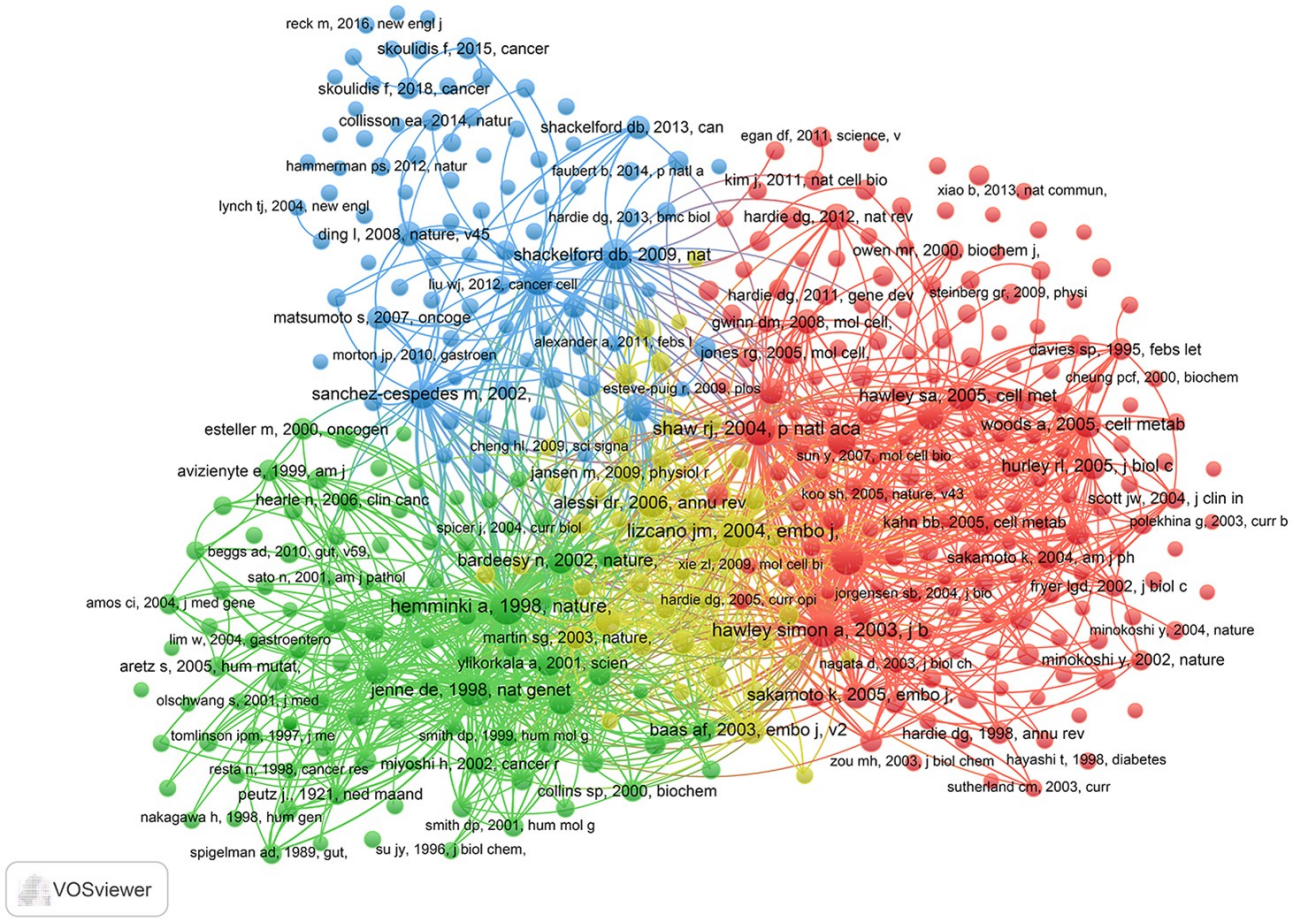
Co-cited references in LKB1 research.

The co-citation analysis of VOSviewer was carried out to analyzed the co-cited references. The top 10 co-cited references were the representative articles of 4 theme clusters. Cluster #1 (red), LKB1 activates AMPK and regulates different biological functions (Shaw RJ, Hawley SA, and Woods A et al.); cluster #2 (green), LKB1 is defective in patients with Peutz-Jeghers syndrome (PJS) (Hemminki A and Jenne DE et al.); cluster #3 (blue), LKB1-AMPK pathway suppresses tumor (Shackelford DB and Ji H et al.); and cluster #4 (yellow), LKB1 related signaling pathways (Lizcano JM and Alessi DR et al.) (Table 5). Fig 7 displays the major co-references and their clusters.

**Table 5:** Top 10 co-cited references in LKB1 research from 2000 to 2021.

Rank	Title	Author (year)	Journal	Citation	Cluster
1	A serine/threonine kinase gene defective in Peutz-Jeghers syndrome.	Hemminki A et al. (1998)	Nature	429	2
2	The tumor suppressor LKB1 kinase directly activates AMP-activated kinase and regulates apoptosis in response to energy stress.	Shaw RJ et al. (2004)	Proceedings of the National Academy of Sciences of the United States of America	408	1
3	LKB1 is a master kinase that activates 13 kinases of the AMPK subfamily, including MARK/PAR-1.	Lizcano JM et al. (2004)	The Embo journal	382	4
4	Complexes between the LKB1 tumor suppressor, STRAD alpha/beta and MO25 alpha/beta are upstream kinases in the AMP-activated protein kinase cascade.	Hawley SA et al. (2003)	Journal of Biology	378	1
5	LKB1 is the upstream kinase in the AMP-activated protein kinase cascade.	Woods A et al. (2003)	Current Biology	367	1
6	Peutz-Jeghers syndrome is caused by mutations in a novel serine threonine kinase.	Jenne DE et al. (1998)	Nature Genetics	306	2
7	The LKB1-AMPK pathway: metabolism and growth control in tumor suppression.	Shackelford DB et al. (2009)	Nature Reviews Cancer	286	3
8	LKB1-dependent signaling pathways.	Alessi DR et al. (2006)	Annual Review of Biochemistry	276	4
9	LKB1 modulates lung cancer differentiation and metastasis.	Ji H et al. (2007)	Nature	251	3
10	The kinase LKB1 mediates glucose homeostasis in liver and therapeutic effects of metformin.	Shaw RJ et al. (2005)	Science	213	1

### Keywords distribution analysis

**Fig 8:**
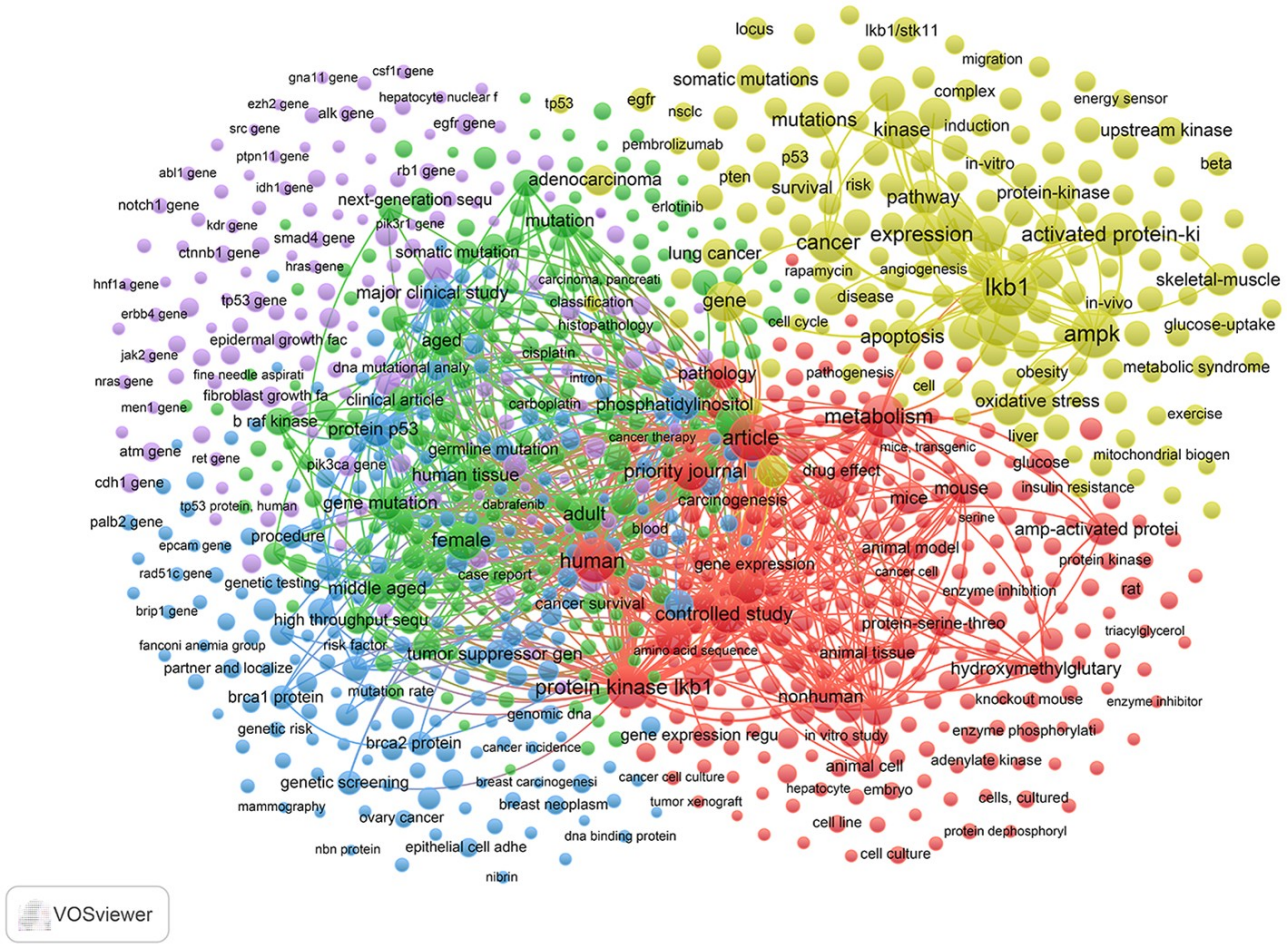
The co-occurrence keywords in LKB1 research.

The 5 clusters of keywords with different research themes were generated by the co-occurrence analysis of VOSviewer. The network visualization map displayed the 5 clusters with the colors of yellow, red, purple, green, and blue, respectively (Fig 8). The top 20 co-occurrence keywords for each cluster are listed in Table 6. The theme of cluster #1 (yellow) can be summarized as the molecular background and biological functions of LKB1. The theme of cluster #2 (red) can be summarized as the expression and molecular functions of LKB1 tested in cells and tissues of animal and human. The theme of cluster #3 (purple) can be summarized as *LKB1* and related genes in tumor. The theme of cluster #4 (green) can be summarized as clinical trials about *LKB1* and related genes mutations in tumors, especially in lung adenocarcinoma. The theme of cluster #5 (blue) can be summarized as co-mutated genes in tumors by gene sequencing. The serial numbers of the 5 clusters were named in order of their average publication time. Cluster #1 were the keywords with the earliest average publication time and the highest co-occurrence frequency, cluster #2 were the keywords with the highest link values, and cluster #4 and #5 were the keywords with the latest publication time. These results reveal that the theme of cluster #1 was the early and mature field of LKB1 research, the theme of cluster #2 was the focused field, and the themes of cluster #4 and #5 are the emerging research fields.

**Table 6:** Top 20 keywords in the 5 clusters of LKB1 research from 2000 to 2021.

Cluster	Top 20 co-occurrence keywords	Average publication year	Average occurrences	Average links
1	lkb1, ampk, expression, activated protein-kinase, phosphorylation, cancer, gene, kinase, growth, activation, apoptosis, mutations, pathway, peutz-jeghers-syndrome, protein-kinase, metformin, cells, protein, oxidative stress, tumor-suppressor	2013.86	304.25	381.05
2	article, human, metabolism, protein kinase lkb1, controlled study, genetics, priority journal, unclassified drug, signal transduction, nonhuman, amp-activated protein kinase, mice, mouse, pathology, animal, human cell, protein expression, hydroxymethylglutaryl coenzyme a reductase kinase, gene expression, animal experiment	2015.64	219.65	771.40
3	stk11 gene, epidermal growth factor receptor 2, oncogene, dna, pten gene, oncogene kras, colorectal cancer, gene frequency, pik3ca gene, fibroblast growth factor receptor 1, tp53 gene, fibroblast growth factor receptor 2, dna mutational analysis, egfr gene, fibroblast growth factor receptor 3, genetic analysis, polymerase chain reaction, atm gene, braf gene, epidermal growth factor receptor 4	2016.63	44.50	541.65
4	female, male, adult, mutation, gene mutation, aged, middle aged, human tissue, epidermal growth factor receptor, kras protein, lung adenocarcinoma, high throughput sequencing, next generation sequencing, braf kinase, prognosis, immunohistochemistry, cyclin dependent kinase inhibitor 2a, cancer staging, clinical article, next-generation sequencing	2017.15	121.15	721.75
5	protein p53, major clinical study, phosphatidylinositol 3,4,5 trisphosphate 3 phosphatase, breast cancer, tumor suppressor gene, apc protein, smad4 protein, atm protein, procedure, brca2 protein, germline mutation, uvomorulin, genetic screening, brca1 protein, mutl protein homolog 1, cancer risk, checkpoint kinase 2, protein msh6, dna sequence, single nucleotide polymorphism	2017.25	76.40	608.85

**Fig 9:**
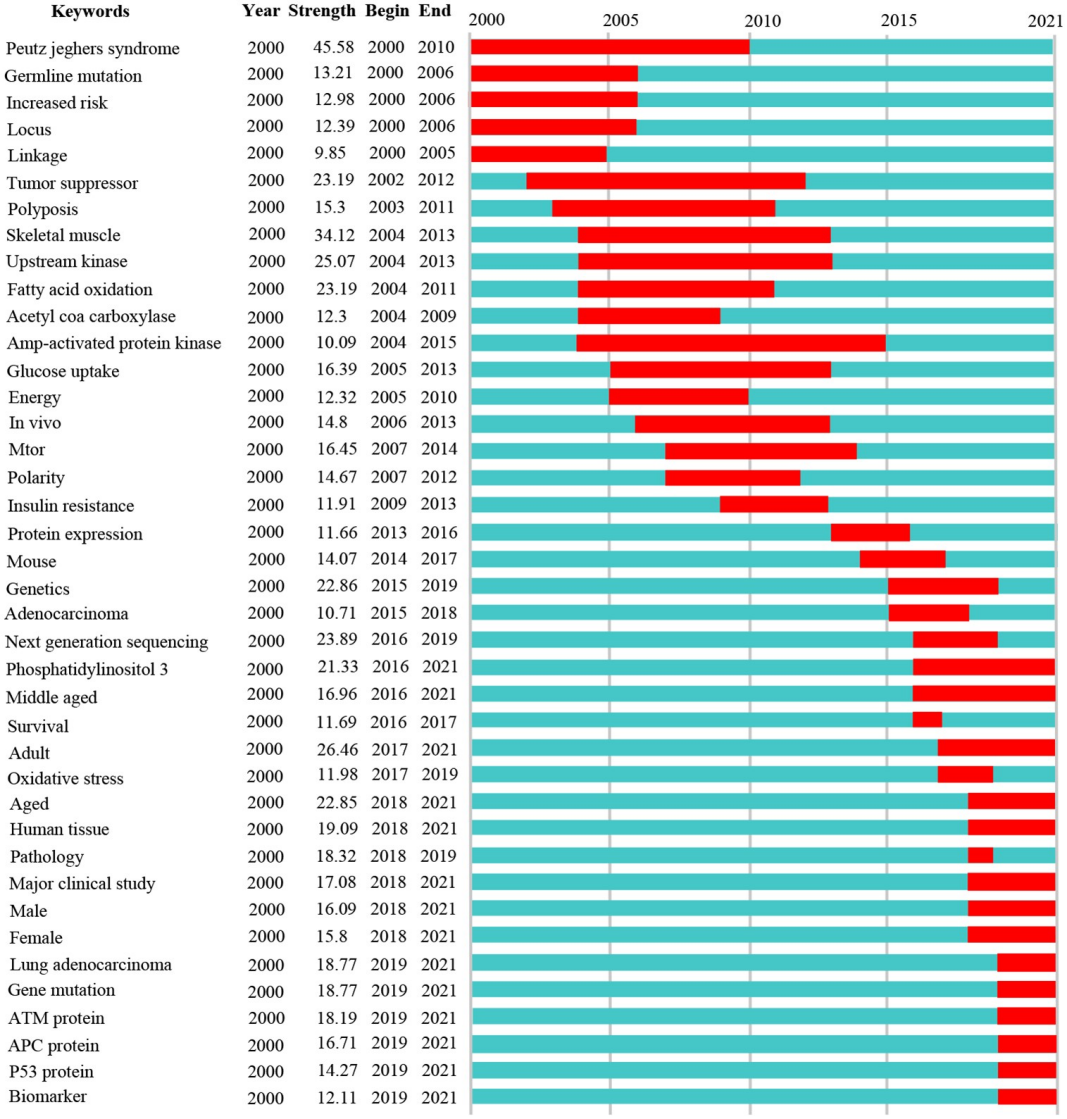
The top 40 strongest citation burst keywords in LKB1 research during 2000–2021. The green bar represents the time interval from 2000 to 2021, and the red bar represents the burst cited time of keyword. “Year” column represents the earliest year in which the keyword had been published in the field of LKB1 research, “Begin” and “End” columns represent the begin and end years between which the keyword had been burst cited, and “Strength” column represents the strength with which the keyword had been burst cited.

We mapped the top 40 burst cited keywords that effectively reflected the evolution of LKB1 research hotspots using CiteSpace (Fig 9). As shown in Fig 9, the development trends of LKB1 research are consistent with the result of co-occurrence keyword analysis of VOSviewer. From 2000 to 2021, the hotspots of LKB1 research shifted from the molecular background and functions to clinical trials and co-mutated genes in tumors. In recent year, the keywords that still maintain the state of bursting citation included “phosphatidylinositol 3, middle aged, adult, aged, human tissue, major clinical study, male, female, lung adenocarcinoma, gene mutation, ATM protein, APC protein, P53 protein, biomarker”, which are considered as emerging hotspots and future research trends of LKB1. The themes of these keywords can be summarized as follows: the clinical studies about *LKB1* and co-mutated genes as biomarkers in tumors, especially in lung adenocarcinoma.

## Discussion

LKB1 was first identified as a tumor suppressor gene in patients with Peutz-Jeghers syndrome (PJS) in 1998 [29, 30], and a large number of publications related to LKB1 have been reported in different fields gradually. In recent years, the potential clinical value of LKB1 has been concerned by scholars. Investigations of LKB1 have important medical implications that require in-depth analysis and summary. Therefore, a better understanding of the current knowledge structure, hotspots, and emerging frontier fields of LKB1 research is crucial for future research. In this study, we conducted a bibliometric analysis of the publications on LKB1 research from 2000 to 2021 for the first time.

In the past 22 years, the number of LKB1 publications has increased gradually by year. In recent years (from 2016 to 2021), the number of publications accounts for nearly half of the total, which indicates that LKB1 has become the focus of attention (Fig 2). Studies on LKB1 are mainly distributed in three disciplinary directions: Oncology, biochemistry molecular biology, and cell biology (Fig 3). Based on our findings, the leading contributors to the LKB1 research were the United States, Harvard University, Benoit Viollet, Journal of Biological Chemistry and Plos One. Meanwhile, more than half of the top 10 publication institutions, authors and journals were from the United States. Therefore, we confirm the dominant role of the United States in LKB1 research. The cooperation of top authors could also reflect the hotspot themes in LKB1 research (Fig 5). The themes of cooperation among Benoit Viollet, D Grahame Hardie, Marc Foretz, Kei Sakamoto, and Dario R Alessi were the molecular functions of LKB1: LKB1 regulates glucose metabolism, fatty acid oxidation, and energy metabolism through activation of AMPK [31–33]. The themes of cooperation among Kwok Kin Wong, Nabeel Bardeesy, and Hongbin Ji were the roles of LKB1 in cancers, such as lung cancer, cervical cancer, and endometrial cancer [3, 34, 35]. The top 10 co-cite references listed in Table 5 are recognized as benchmarking publications, which also represent the hotspots of LKB1 research. Combined with the results of the co-authorship authors and co-cited reference analysis, the shared hotspots of LKB1 were as following: LKB1 regulates different biological functions through AMPK activation, and the roles that LKB1 plays in cancer. However, the details of the hotspots and the evolution process could not be accurately illustrated by the present results. Therefore, we conducted keywords analysis to discover the evolution of hotspots and predict the future trends. Combined with the results of co-occurrence and burst keywords analysis, we found that the focused hotspot was the molecular functions of LKB1, and the emerging hotspots are the clinical studies about *LKB1* and co-mutated genes as biomarkers in tumors, especially in lung adenocarcinoma. These emerging hotspots can also be considered as the future research trends.

Our results reveal that the molecular biological functions of LKB1 was the focused hotspot. LKB1, a well-characterised protein kinase, localises mainly in nuclei. LKB1 binds to the pseudokinase, STRADα, and the scaffolding protein, MO25, cause it to relocate to the cytoplasm, as well as enhancing its kinase activity [36–38]. In different cellular environments, LKB1 is considered as a master kinase to activate 14 AMP-activated protein kinase (AMPK) family members [39–42] and non-AMPK family proteins, such as LIP1, PTEN, and p53-p21/WAF1 [43–45], so as to regulate different molecular biological functions. Co-occurrence and citation burst keywords analysis showed that oxidative stress, metabolism, insulin resistance, apoptosis, and cell polarity were the hotspots of molecular biological functions. In the function of mediating oxidative stress, loss of LKB1 expression has been shown to increase reactive oxygen species (ROS) levels, leading to accumulate DNA damage of cancer cells, raising the sensitivity of cancer cells to oxidative stress inducing therapies such as cisplatin and γ-irradiation [46, 47]. The dominant academic view supports that the role of LKB1 in oxidative stress depends on AMPK [46, 48, 49]; however, different view supports that the role of LKB1 in suppressing ROS is independent of AMPK [47]. Besides, as the central metabolic sensor, AMPK is activated by LKB1 to regulate various metabolic progresses, such as energy, glucose and lipid metabolism [2]. In skeletal muscle, LKB1 and AMPK enhance glucose transport, lipid and fatty acid oxidation, and insulin sensitivity, and may, therefore, be treatment targets for type 2 diabetes and obesity [50, 51]. Depending on metabolism or ROS, LKB1-AMPK pathway can induce autophagy [52, 53], and autophagy deficiency can inhibit the proliferation of LKB1 deficient lung cancer cells by regulating lipid metabolism [54]. Under energy shortage conditions, the LKB1-AMPK axis suppresses cancer cell proliferation by inhibiting fatty acid and protein synthesis, as well as glycogen storage [55, 56]. Earlier studies have found that LKB1 regulates apoptosis depending on p53-dependent pathways [57]. LKB1 requires SIK1 (an AMPK family member) to promote p53-dependent anoikis, a form of apoptosis caused by poor contact between the cell and the extracellular matrix, so as to suppress cell growth and invasion [58]. LKB1 also can inhibit cell growth through suppressing the anti-apoptotic factors, such as STAT3, JNK, KRAS, MAPK, cyclooxygenase-2, and c-myc [42, 59, 60]. In recent studies, LKB1-AMPK is still the major signaling pathway to regulate cell apoptosis [61–63]. The role of LKB1 plays in epithelial polarity is associated with MARK/PAR1 and AMPK. LKB1 phosphorylates MARK/PAR1 kinases, which is associated with cell polarity regulated by LKB1 [64, 65]. LKB1 coordinates epithelial polarity and proliferation according to cellular energy status through AMPK [66]. There are some evidences support that LKB1-AMPK pathway may promote tumorigenesis by maintaining metabolic homeostasis and preventing oxidative stress [67–69]. The role of LKB1 in cell polarity and metabolism is dual, besides suppressing tumorigenesis, it main also promotes tumor development. LKB1 regulates epithelial polarity to promote tumorigenesis through inactivating class III phosphatidylinositol-3-OH kinase (CIII-PI3K) [70]. It can be concluded from the above discussion that the biological functions of LKB1 are interdependent and interactive in the development of metabolic diseases and tumors.

According to the results of bibliometric analysis, the clinical studies about *LKB1* and co-mutated genes as biomarkers in tumors, especially in lung adenocarcinoma, are the emerging hotspots and future trends. *LKB1* was first identified as a tumor suppressor gene in patients with Peutz-Jeghers syndrome (PJS), a rare autosomal dominant disorder characterized by the growth of multiple hamartomatous gastrointestinal polyps, pigmented mucocutaneous macules, and other neoplasms [29, 30]. Approximately 94%–96% of patients with PJS have germline mutations of *LKB1*, which is associated with 10-fold higher cancer risk than that of the general population [71, 72]. Soon after the identification of germline *LKB1* mutations in PJS, *LKB1* somatic mutations were detected as associated with poor survival of patients with sporadic malignancies, such as non-small cell lung cancer (NSCLC), breast cancer, pancreatic cancer, colon cancer, cervical cancer, and melanoma [35, 73–81]. Especially in NSCLC, *LKB1* has the third highest mutation rate of approximately 34%, second only to *TP53* and *KRAS* [3, 73]. The *LKB1* mutation rates in lung squamous cell carcinoma and large cell carcinomas are about 19% and 14% [82]. Mutations of *LKB1* frequently co-occur with *KRAS* and *TP53* mutations in NSCLC, which are associated with a higher risk of metastasis and poor prognosis compared with *KRAS* or *TP53* mutation alone [83, 84]. As a tumor suppressor gene in NSCLC, LKB1 regulates AMPK, mTOR, VEGF, p53, p21/WAF1, SIK1, SIK3, and INSL4 to inhibit cell proliferation, cell differentiation, cell invasion, cell migration, tumor angiogenesis, and cell cycle arrest [85–89]. Further, LKB1 inactivation induces a redox imbalance to promote transdifferentiation from lung adenocarcinoma to lung squamous cell carcinoma in NSCLC, which leads resistance to anti-tumor therapy [90]. Therefore, the prognosis of patients with *LKB1* mutated NSCLC has been the focus of substantial attention; and related therapeutic clinical trials, requiring *LKB1* mutation as a determinant or investigation inclusion criteria, have been conducted. LKB1 has been proven to be the most prevalent driver gene of resistance to PD-1 inhibitor in *KRAS*-mutant lung adenocarcinoma [16], while in *LKB1*-mutant non-squamous non-small cell lung cancer (mnsNSCLC), pembrolizumab did not improve the PFS and OS of patients administered platinum-pemetrexed chemotherapy [91]. In patients with advanced LKB1-inactive NSCLC receiving platinum-pemetrexed chemotherapy, although metformin had been administered first, it could not improve prognosis in a phase II clinical trial, due to limited sample numbers [15]. Another phase II clinical trial (NCT03709147) will begin recruiting to evaluate the clinical outcomes of treatment with metformin combined with fasting mimicking diet. In addition, a phase III clinical trial to evaluate the disease control rate of talazoparib and avelumab for patients with stage IV or recurrent mnsNSCLC with *LKB1* mutation are currently recruiting (NCT04173507). Since *LKB1* and *KRAS* mutations in tumors are still considered as undruggable targets, genetic aberrations screening of clinical tumor specimens is being carried out gradually. A phase I trial reported that in addition to *KRAS* and/or *TP53* mutations, the most common concurrent genetic aberrations in NSCLC were *CDKN2A*, *EGFR*, *BRAF*, *PIK3CA*, *ATM*, *APC*, *STK11*, *c-MET* and *KIT* [92]. With the wide application of next generation sequencing, the co-mutation genes in different tumors were detected, such as, the co-mutation of *TP53*, *STK11*, *CDKN2A* and *KMT2C* in lung cancer; the co-mutation of *TP53*, *KRAS*, *ARID1A*, *PIK3CA*, *CDKN2A*, *SMARCA4*, *PBRM1*, *STK11*, *APC* and *RB1* in cancer of unknown primary; the co-mutation of *ABCC12*, *APC*, *ATM*, *BRCA1*, *BRCA2*, *CDH1*, *ERCC6*, *MSH2*, *POLH*, *PRF1*, *SLX4*, *STK11* and *TP53* in breast cancer; and the co-mutation of *KRAS*, *GNAS*, *AKT1*, *APC*, *PIK3CA*, *RB1*, *STK11* and *TP53* in low-grade mucinous neoplasms [93–96].

Although the analytical methods used in this study can describe the core power and hotspot evolution of LKB1 research, the publications of LKB1 have not been comprehensively analyzed due to certain limitations. The data we analyzed were extracted from WoSCC, Scopus, and PubMed, but did not include Embase, Google Scholar and other databases; hence, our data may not be representative of all LKB1 studies. However, the data offered by the three databases covers the overwhelming majority of publications in LKB1 field. Furthermore, although our retrieval strategy did not limit language, most publications are in English, hence there may have been a linguistic bias.

## Conclusions

In conclusion, through bibliometric visualization analysis, the core power and hotspot evolution of LKB1 research are visually displayed. In the past 22 years, the number of publications on LKB1 has increased steadily. The United States exerted an important influence on LKB1 field. Frequent and effective cooperation between countries, institutions and authors is beneficial for promoting LKB1 research. The focused research hotspot was the molecular functions of LKB1. The emerging hotspots and future trends are the clinical studies about *LKB1* and co-mutated genes as biomarkers in tumors, especially in lung adenocarcinoma. We conclude that multi-target joint surveillance and intervention may be the mainstream direction of future clinical research on LKB1 field.

## Supporting information

S1 File Retrieval strategies for LKB1 related publications in three databases. (DOCX)

S1 Dataset LKB1 related publications from three public databases. (TXT)
