# Supplementary material for: Hotspots and trends in liver kinase B1 research: A bibliometric analysis
Source: PLoS One. 2021 Nov 4;16(11):e0259240. doi: 10.1371/journal.pone.0259240 (PMC8568265; doi:10.1371/journal.pone.0259240)
Supplement: S1 File — (DOCX) [file pone.0259240.s001.docx]

Web of Science:

TI=(((LKB1) OR ("liver kinase B1")) OR (STK11)) OR ("serine-threonine kinase 11")

Document type: “article”

Year range: “2000 to 2021”

Search date: June 1, 2021.

Result: 2665

Scopus:

( TITLE-ABS-KEY ( "LKB1" )  OR  TITLE-ABS-KEY ( "STK11" )  OR  TITLE-ABS-KEY ( "liver kinase B1" )  OR  TITLE-ABS-KEY ( "serine-threonine kinase 11" ) )  AND  ( LIMIT-TO ( PUBYEAR ,  2021 )  OR  LIMIT-TO ( PUBYEAR ,  2020 )  OR  LIMIT-TO ( PUBYEAR ,  2019 )  OR  LIMIT-TO ( PUBYEAR ,  2018 )  OR  LIMIT-TO ( PUBYEAR ,  2017 )  OR  LIMIT-TO ( PUBYEAR ,  2016 )  OR  LIMIT-TO ( PUBYEAR ,  2015 )  OR  LIMIT-TO ( PUBYEAR ,  2014 )  OR  LIMIT-TO ( PUBYEAR ,  2013 )  OR  LIMIT-TO ( PUBYEAR ,  2012 )  OR  LIMIT-TO ( PUBYEAR ,  2011 )  OR  LIMIT-TO ( PUBYEAR ,  2010 )  OR  LIMIT-TO ( PUBYEAR ,  2009 )  OR  LIMIT-TO ( PUBYEAR ,  2008 )  OR  LIMIT-TO ( PUBYEAR ,  2007 )  OR  LIMIT-TO ( PUBYEAR ,  2006 )  OR  LIMIT-TO ( PUBYEAR ,  2005 )  OR  LIMIT-TO ( PUBYEAR ,  2004 )  OR  LIMIT-TO ( PUBYEAR ,  2003 )  OR  LIMIT-TO ( PUBYEAR ,  2002 )  OR  LIMIT-TO ( PUBYEAR ,  2001 )  OR  LIMIT-TO ( PUBYEAR ,  2000 ) )  AND  ( LIMIT-TO ( DOCTYPE ,  "ar" ) )  AND  ( LIMIT-TO ( PUBSTAGE ,  "final" ) )

Search date: June 1, 2021.

Result: 3089

PubMed:

("LKB1"[Title/Abstract] OR "liver kinase B1"[Title/Abstract] OR "STK11"[Title/Abstract] OR "serine-threonine kinase 11"[Title/Abstract]) AND ((journal article [Filter]) AND (2000/1/1:2021/6/1[pdat]))

Search date: June 1, 2021.

Result: 2888
